# Accessing Responsible Gambling Information from Casinos: Two Secret Shopper Studies

**DOI:** 10.1007/s10899-025-10396-w

**Published:** 2025-05-24

**Authors:** Brianna Morelli, Margaret Anne Gunnigle, Lilia M. Russell, Chance V. Dow, Julia G. Schuetze, Meredith K. Ginley, James P. Whelan, Rory A. Pfund

**Affiliations:** 1 Department of Psychology, University of Memphis, Memphis, TN USA; 2 Department of Psychology, Rhodes College, Memphis, TN USA; 3 Department of Psychology, East Tennessee State University, Johnson City, TN USA; 4 Tennessee Institute for Gambling Education and Research, Memphis, TN USA

**Keywords:** Casino, Responsible gambling, Secret shopper, Corporate social responsibility

## Abstract

Although responsible gambling (RG) programs are prevalent as they are legally required in many jurisdictions and are commonly found as part of corporate social responsibility policies, consumers report barriers to and limited employee knowledge about RG resources. Using a secret shopper methodology, two studies explored the accessibility of information about casino RG programs. The two studies sampled all seven casinos located within a southeast United States metropolitan area of 1.3 million people. Study 1 involved contacting each casino by telephone and requesting information from customer services representatives (*n* = 12), and Study 2 involved visiting casinos in person and requesting information from on-casino floor employees (*n* = 6), off-casino floor employees (*n* = 7), and security personnel (*n* = 7). The information requested through both modalities included (1) general information about RG, (2) the presence of RG resources both in-casino and online, and (3) directions to find RG resources in-casino and online. Study 1 indicated that the accessibility of RG information was unreliable via the telephone. Study 2 indicated that casino employees provided information about RG and in-casino materials but inconsistently provided information about online RG resources. These two studies suggested that casinos could not reliably provide comprehensive RG information. This finding suggests a greater need for governments to hold operators accountable for offering RG programs and for operators to better adhere to corporate social responsibility commitments.

**Supplementary Information:**

The online version contains supplementary material available at 10.1007/s10899-025-10396-w.

In 2023, the United States (US) commercial gaming revenue broke the annual record for the third year, totaling $66.5 billion (American Gaming Association, 2024). Despite the increasing attention to online gambling, 75% of this revenue originated from land-based casino gambling (American Gaming Association, 2023). US-based casino gambling often brings to mind large markets such as Las Vegas, Nevada, and Atlantic City, New Jersey, the casino markets with the first and second highest revenues in 2023, combining for $11.7 billion (American Gaming Commission, 2023). However, this revenue only comprised 23% of the total land-based gaming revenue in the US, with the remaining revenue ($39 billion) produced in small markets near moderate-sized metropolitan areas (American Gaming Commission, 2023). Since 1976, the prevalence of small markets expanded access to casino gaming nationwide (Fenich, 2012).

A recent review reported that 60–65% of US adults gambled in the past twelve months (Tran et al., 2024). Although most who gamble do not encounter significant harm, 14% engaged in risky gambling and 2% met the threshold for diagnosable conditions (Tran et al., 2024). These harms occur when individuals’ wagering impedes personal, familial, and economic aspects of life (Langham et al., 2016). In response to concerns about gambling harm, industry operators have incorporated Corporate Social Responsibility (CSR) initiatives into their business practices, which are strategies that inculcate social and community concerns into business operations (Kim et al., 2017; Tetrevova, 2023). Coherent CSR strategies enhance a business’s positive impact on society (Berkowitz & Daniels, 1964; You, 2024). For the gaming industry, CSR strategies should include customers’ ready access to educational resources on the risks associated with gambling. These CSR consistent protections ideally encourage what is referred to as responsible gambling (RG; Blaszczynski et al., 2022).

Consistent with CSR, most US jurisdictions have mandated operators to offer RG programs (American Gaming Association, 2023). These regulations have included the provision of practical and easily consumed informational material for customers, as well as employee training about RG programs (American Gaming Association, 2023). Casinos have also educated employees about gambling harms, accessible customer resources, and how to respond to customer requests (LaPlante et al., 2012; Shaffer et al., 2019; Hing & Nuske, 2012). Furthermore, regulations have required casinos to make RG information (e.g., pamphlets, helpline numbers) physically available at different locations, such as the cashier desk or on automated teller machines (Mississippi Gaming Control Act, 1990, 2020; Game of Skill Act, 2005). In jurisdictions where RG programs have not been mandated, operators have been recommended to voluntarily provide such programs (American Gaming Commission, 2023).

Despite the accessibility of RG programs, customers have encountered barriers to accessing RG information and have reported casino employees have limited knowledge of venue-specific RG resources (Gainsbury, 2014; Hing et al., 2014; Hing & Nuske, 2011a; Ladouceur et al., 2017; Pickering et al., 2019). Past research has also explored employee responses to customer requests for assistance (Hing & Nuske, 2011a, b; Riley et al., 2018, 2023), and some employees have reported feeling uncomfortable with offering RG programs to customers (Beckett et al., 2020; Hing & Nuske, 2012; Riley et al., 2018). Left unexplored is whether venues effectively provide customers with RG information when requests are made.

The current paper includes two studies that capture the experience of customers’ pursuit of RG information within land-based casinos. The casinos were located in a southeastern market serving a moderate-sized metropolitan area of approximately 1.3 million people (Census Profile, 2023). Six of the seven casinos contacted and visited in this project are within a jurisdiction that legally requires operators to train employees on problem gambling, including helping customers with “obtaining information about problem gambling programs” (Mississippi Gaming Control Act, 1990, 2020). These regulations also require operators to “provide at each entrance and exit to the gaming premises, and in conspicuous places in or near gaming or cage areas and cash dispensing machines located on the gaming premises written materials concerning the nature and symptoms of problem gambling” (Mississippi Gaming Control Act, 1990, 2020). The regulations for the seventh casino, which is located in a different legal jurisdiction, require operators to “conspicuously display a responsible gambling message, which includes a helpline and/or website that can provide problem gambling information and assistance” (Game of Skill Act, 2005). All seven property websites contain information about and pledge a commitment to RG. For example, the website for one casino includes the following language: “We communicate a responsible gaming message daily to our guests both verbally and in printed mediums throughout our facility” (*Responsible Gaming in AR - Southland Casino*, n.d.).

Using a secret shopper methodology (Rankin et al., 2022), two studies were conducted where investigators posed as customers seeking RG information to explore customer experiences in accessing RG information. Study 1 involved using casinos’ advertised customer service numbers to request RG information, whereas Study 2 involved in-person visits to the same casinos to request RG information. Since RG programs were legally required of six casino operators and promised through CSR commitments by one operator, it was hypothesized that employees would successfully execute their mandated or promised responsibility to provide the requested information.

## Study 1

This study explored customer experiences during telephone calls to casino customer service numbers requesting information about how to access RG information. The university’s Institutional Review Board determined this study was not human subjects research, as it explored interactions with businesses rather than individuals.

### Method

The study design was adapted from past secret shopper studies (Kilcommons et al., 2020; Uscher-Pines et al., 2023; Meyerson et al., 2024) and guidelines (Rankin et al., 2022). Casinos did not receive any prior notification of these calls.

#### Procedure

A script was created to request RG information from casinos. The script included questions regarding general information about RG programs and whether the information was available online and physically available at the casino. The script closed with a request for a mailed hard copy of the RG information (See Supplemental Material 1). Research assistants were trained to complete these calls in a comfortable, relaxed voice and with fidelity to a script. This competence was achieved by using practice phone calls. Research assistants were also trained to respond to questions they might receive during the call. All calls were made to casinos’ web-accessed and operable customer service numbers during the summer of 2024 during standard business hours. Casinos were randomly assigned to customers using a random number generator, and no customer called a specific casino more than once. All casinos were called twice to assess whether casino customer service staff provided consistent information. Research assistants will be referred to as “customers” in the remainder of this paper.

Customers completed caller experience forms for each completed phone call. The duration of the call and time spent on hold were recorded. Customers also recorded whether they received any information related to avoiding harmful gambling or gambling within affordable limits. Customers rated their subjective experiences with the casino using four seven-point Likert scale questions. Questions assessed employee attentiveness (1 = *very dismissive*, 7 = *very attentive*), demeanor (1 = *very judgmental*, 7 = *very accepting*), friendliness (1 = *very unfriendly*, 7 = *very friendly*), and knowledge of RG (1 = *very ill-informed*, 7 = *very knowledgeable*). RG knowledge was defined as the ability to provide accurate answers to the questions in the script (e.g., awareness of online and in-casino resources), and to provide information consistent with the definition of RG (e.g., not gambling in a harmful way or gambling within affordable limits; Blaszczynski et al., 2022). Immediately following the phone call, customers noted pertinent customer service experience details.

#### Data Analysis Plan

Using Statistical Package for Social Sciences (SPSS; Version 29), descriptive statistics were computed for call duration, time spent on hold, and the number of employee interactions. Frequency analyses were computed with binary variables (e.g., Yes or No) to determine whether employees provided general information about RG, indicated the physical presence of information in their venue, and the presence of information online. Frequencies were also calculated for whether employees provided directions about where to find the information in their physical venue and on their website, whether RG information could be mailed, and whether the RG information was received in the mail. Frequency analyses were computed for each of the four 7-point Likert scale questions. Comparisons between the first and second calls to each casino were used to assess the reliability of the information provided.

### Results

#### Time Spent Requesting Information

Across all 12 telephone calls, the average call length was 5 min (*SD* = 3.13, range = 1.6 to 10), and the average time on hold during the call was 2 min (*SD* = 2.2, range = 0.12 to 6). On average, customers spoke to 2 employees (*SD* = 0.08; range = 1 to 4) during the call (See supplemental Table 1 for call information).

#### Information Provided by Casinos

**Fig. 1:**
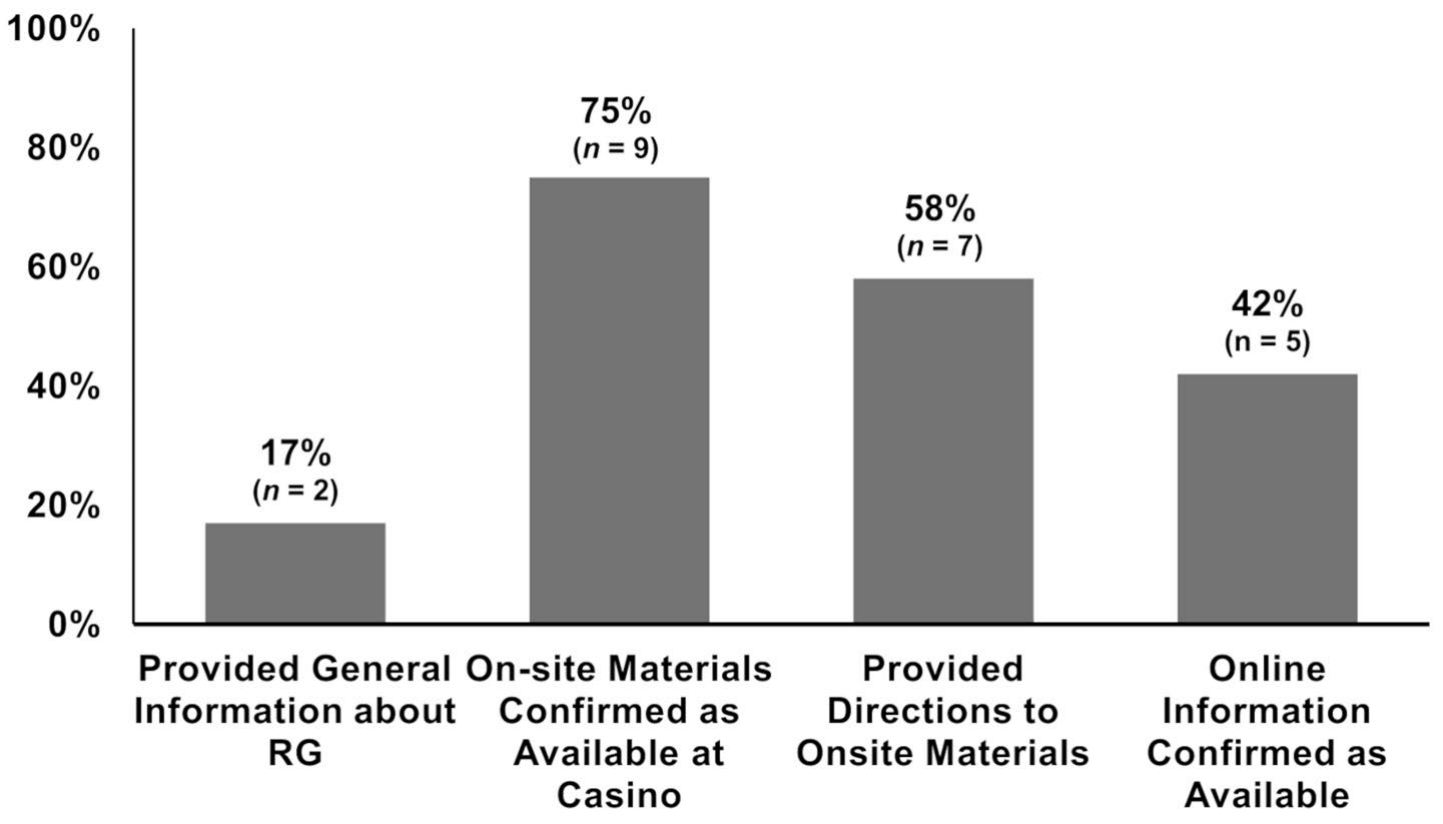
Percentage of telephone calls (*n* = 12) that correctly provided requested RG information Note: This graph displays the percentage of calls where employees were able to provide the requested responsible gambling information. General information refers to information about safe gambling practices. Callers asked employees questions regarding general explanations of responsible gambling, the existence of in-casino materials, and if information was available online. Six casinos were called twice, resulting in 12 total calls

Figure 1 presents the percentage of calls that provided RG information. Of 12 customer services representatives, only two (17%) conveyed any general understanding of RG. However, 75% (*n* = 9) indicated that RG information was physically available at the casino, and 58% (*n* = 7) provided a location for such materials. As for online materials, 41% (*n* = 5) correctly indicated that information was on the website, but only 17% (*n* = 1) could report where the information was located on the website. All customer service representatives said that RG materials could not be mailed.

#### Information Reliability

**Fig. 2:**
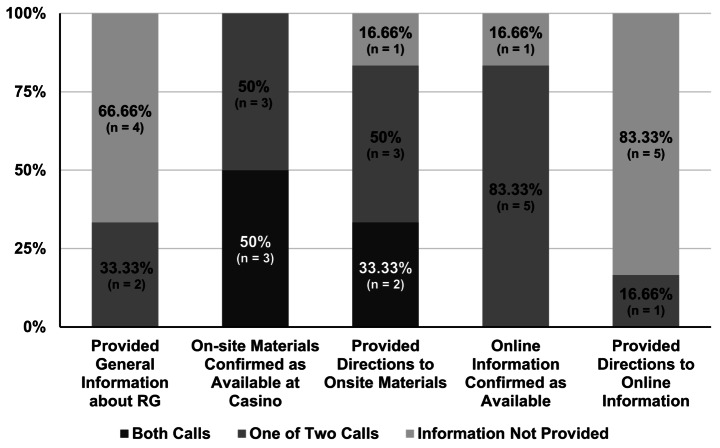
Percentage of casinos (*n* = 6) that provided the requested RG information in both, one, or neither call Note: This figure demonstrates the percentages of casinos that provided information on responsible gambling. A total of six casinos were called twice, and casinos were assessed on the consistency of their answers. Two calls to each property were compared to assess if the same information was provided

No casino reliably provided general RG information during both calls. 33% (*n* = 2) provided general information during one of two calls, and 66% (*n* = 4) did not provide general information during either call (see Fig. 2). When asked if RG information was available at the casino, 50% (*n* = 3) affirmed availability during both calls to the same casino, and 50% (*n* = 3) affirmed the existence of in-casino materials during only one call. No casino affirmed the existence of online materials during both calls. Five of six casinos (83%) affirmed that there was RG information on their website during one of two calls, and 17% of casinos (*n* = 1) could not affirm that the website included RG information during either call.

#### Customer Service Experience

Table 1 displays the ratings and summaries from each call to each casino property. Generally, employee attentiveness was perceived negatively, with customers typically rating the call as somewhat dismissive and neutral (*M* = 3.2, *SD* = 1.4). 41% (*n* = 5) of customers rated the employees as being at least somewhat dismissive, and 50% (*n* = 6) were rated as neutral. Both customers who called Casino #6 rated the calls as very dismissive. One felt the call was rushed, and the other reported that the employee hung up the phone after being asked if RG materials could be mailed to the customer.

**Table 1:** Likert scale ratings and narrative summaries from the first and second call to each casino property

Casino #	Call	Ratings	Summary
Casino #1	1st	Dismissive Somewhat Judgmental Somewhat Unfriendly Somewhat Ill-informed	First employee responded with “what?” when I asked about brochures/pamphlets. Second employee said that they would be at the cashier cage. When I asked about online materials, the employee seemed very annoyed and said “I don’t know if anything is online, probably”. Overall just felt like they didn’t want to talk to me
Casino #1	2nd	Dismissive Somewhat Judgmental Somewhat Unfriendly Somewhat Ill-informed	The first employee transferred me to the second employee because she said the second employee might have better information. The second employee listened to my question about gambling pamphlets (I think I also had an internet issue at this point because she couldn’t hear me) then she said to hold on and I was put on hold for around 5 min. I think she was confused about what I meant about general information about responsible gambling and told me that there was information on their site. Based off her tone when talking about there being information on their site, I think she didn’t understand why I was calling and didn’t just look on their website. She did confirm there were pamphlets online and in the casino. When I asked about mailing pamphlets, she said they don’t do that and hung up shortly after.
Casino #2	1st	Neutral Neutral Somewhat Unfriendly Somewhat Ill-informed	I didn’t feel like the employee really knew what to say about most things. When asked about directions online she said that I could just go on their website and find the materials. It seemed like she was trying to get me off the phone. She took an uncomfortably long pause to answer my question about where the materials were in the casino.
Casino #2	2nd	Neutral Somewhat Accepting Somewhat Friendly Ill-informed	This was a very short interaction. I was connected to someone pretty fast who then put me on hold and transferred me to someone else immediately when I asked her if she knew any general information. When I asked the second employee about general information she and was hesitant and nervous to answer and told me she could leave my name and number for a helpline to get back to me. I also tried asking about materials in the casino, she was apologetic like she wanted to help but it seemed like she wanted to end the call because she didn’t have information.
Casino #3	1st	Neutral Neutral Neutral Ill-informed	I would say about 75% of this call was spent on hold. The first employee I spoke to put me on a brief (over 2 min hold) then gave me the number for the casinos responsible gaming helpline and said that they do have brochures in the casino. When I asked if he knew where the brochures were in the casino he transferred me to another employee, who transferred me again. The last woman I spoke to said she was transferring me to the casino chief manager, but after a minute on hold I was hung up on.
Casino #3	2nd	Neutral Neutral Neutral Somewhat Ill-informed	The call was short, and the employee was very neutral. She talked about self-exclusion exclusively and when I asked about responsible gambling materials and if there are some online or in the casino, she gave me a number to call and ask them. XXX-XXX-XXX
Casino #4	1st	Neutral Somewhat Accepting Neutral Neutral	When I first asked she immediately put me on hold to give me a hotline number. When I asked about materials in the casino she gave me directions. After following up, she said to call the hotline for any more information.
Casino #4	2nd	Neutral Neutral Neutral Somewhat Ill-informed	It didn’t seem like any of the employees knew what responsible gambling was.
Casino #5	1st	Attentive Accepting Very friendly Somewhat Knowledgeable	While I think the woman didn’t hear me fully at first or just didn’t understand what I was asking, she did give me beneficial information both about the casino in general and about where the responsible gambling materials are. She could have provided me more information, yes, but she was so nice and never got upset with me when I asked more questions.
Casino #5	2nd	Somewhat dismissive Neutral Neutral Somewhat Ill-informed	The call was very quick. She asked what in particular I wanted to know about responsible gambling and asked if I wanted to know about self-exclusion. I asked for general information and she just listed the resources in the casino, online, and gave me the hotline number.
Casino #6	1st	Very dismissive Somewhat Judgmental Somewhat Friendly Somewhat Ill-informed	When I asked about responsible gambling materials, she said “uh what? Hold on I don’t know let me send you to someone else” next person told me there were brochures at the cashier, and when I asked if she could mail them she put me on hold and then gave me a problem gambling number (XXX-XXX-XXX) Then I asked again can you mail anything and she said no. When I asked about info online, she said she just didn’t know. After she gave me the number she was really rushing to end the call.
Casino #6	2nd	Very Dismissive Neutral Very Unfriendly Ill-informed	The first employee immediately transferred me when I asked about materials. The second employee was extremely dismissive and told me they had no materials in the casino. She told me to go to the DEIDENTIFIED gaming commission website, then hung up on me immediately after I tried to confirm that she could not mail me any materials.

Perceptions of judgment or acceptance were neutral (*M* = 4, *SD* = 0.9). However, 25% (*n* = 3) were rated as somewhat judgmental. Both customers who called Casino #1 indicated the call felt somewhat judgmental, with one customer reporting that they felt like the customer service representative did not want to speak to them.

As for friendliness, customers experienced the call as neutral (*M* = 3.9, *SD* = 1.4). 25% (*n* = 3) were perceived as being somewhat unfriendly, with one customer reporting that they felt like the customer service representative at Casino #2 wanted them to end the call. A customer who called Casino #1 reported that the customer service representative seemed “annoyed” with them after requesting information. However, one customer rated the experience with Casino #5 as very friendly despite not receiving much information about RG.

In terms of knowledge, ratings were negative, with customers often judging the call as somewhat ill-informed (*M* = 3.0, *SD* = 0.8). 83% (*n* = 10) of calls were rated as ill-informed and somewhat ill-informed, with customers across different casinos reporting that customer service representatives did not understand anything about RG. After speaking with two representatives from Casino #3, one customer was told they were being transferred to the casino chief manager for more information. Instead, the call was disconnected.

## Study 2

This study explored accessing RG during casino visits. As with Study 1, the university’s Institutional Review Board determined that this study was not human subjects research.

### Method

Using the secret shopper methodology, trained research assistants visited the same seven casinos referenced in Study 1, posing as customers seeking RG information. An actor-observer arrangement explored the requests from two perspectives, where the actor engaged with the employee and the observer watched from a moderate distance. Casinos did not receive any prior notification of these visits. At each property, three employees were approached: a casino floor employee (on-floor), a non-casino floor employee (off-floor), and a casino security officer. It was anticipated that all employees would support pursuing RG information, with off-floor employees being the least familiar with RG and on-floor employees being the most familiar. For the remainder of this paper, the trained research assistants will be referred to as “customers”.

#### Procedure

The Study 1 script was modified for in-person interactions. Two customers above the age of 21 were trained to complete the interactions in a confident yet inquisitive manner. Research team members practiced the script with customers to ensure that they were familiar with the questions and had confidence in their delivery. When the customers were able to present the scripted content from memory, the casino visits were scheduled. The script included requests for general information about RG, whether this information was available online and at the casino, and directions for finding the materials (See supplementary materials for full script).

All seven casinos were visited during the summer of 2024 during daytime weekend hours. Casinos were visited in a randomized order. Before entering each casino, customers were randomly assigned to the role of actor or observer, and the sequence of approaching each employee was also randomized.

Upon entering the casinos, customers searched for and documented the presence of RG information at expected locations (e.g., cashier desk, player services). Next, the research team approached the first available employee who fit the randomized role sequence and inquired about RG. Following this conversation, customers walked a reasonable distance away from the employee before using their phones to independently record their experiences about securing information related to not gambling in a harmful way or gambling within affordable limits, if they received correct directions to informational material, and any reactions to their experience.

#### Data Analysis Plan

Analyses were conducted using Statistical Package for the Social Sciences (SPSS), version 29 (IBM Corp., 2022). Actor and observer data were considered separately. A multiple response crosstabulation was conducted for the reception of general information, employee reports of the presence of on-site and online informational material, and directions to on-site and online material for each category of employee. A frequency analysis was conducted for the information noted above, as well as the 7-point Likert scales used in Study 1.

### Results

At one casino, no floor employee appeared available to interact with the customer, and after waiting a reasonable amount of time, this interaction was omitted. Consequently, 20 of the targeted 21 interactions were completed.

#### Location of RG Material

All casinos had RG pamphlets in multiple locations, and they were all considered readily accessible. 71% (*n* = 5) had pamphlets displayed on ATMs, 85% percent (*n* = 6) offered pamphlets on cashier desks, and 80% (*n* = 4) provided pamphlets at the player services desk.

#### Information Received: Actor

**Fig. 3:**
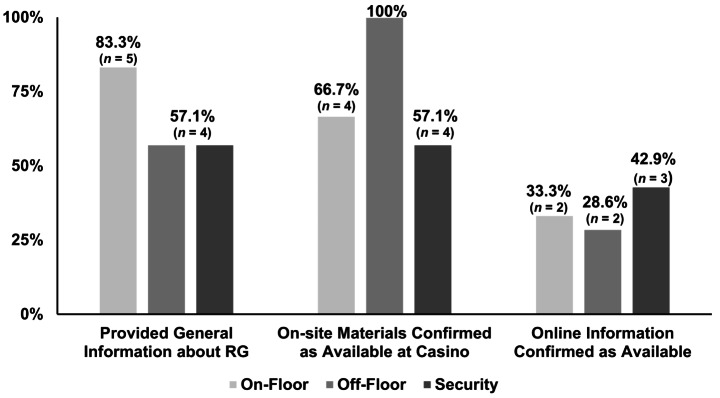
Actor’s experience of whether on-floor (*n* = 6), off-floor (*n* = 7), and security (*n* = 7) employees provided requested information Note: The figure presents the information recieved from casino employees. Actors approached three employees at seven different casinos and asked questions regarding general information about RG, informational materials present on-site, and availability of online information. One on-floor employee could not be located, resulting in a total of 20 employees that were approached for information. The graph displays information gained from three different employees at each casino property

Figure 3 presents the percentage of employees in each of the three casino roles (e.g., on-floor, *n* = 6; off-floor, *n* = 7; security, *n* = 7) that correctly provided the requested information. 83% (*n* = 5) of on-floor employees verbally provided RG general information; 67% (*n* = 4) reported that RG material was present within the casino, and 67% provided accurate directions to the material; 33% (*n* = 2) reported that RG information was present online with only 17% (*n* = 1) indicating how to find the online information.

57% (*n* = 4) of off-floor employees provided general information. Every off-floor employee affirmed that material was present within the casino, and 86% (*n* = 6) provided accurate directions to find the materials. In contrast, only 29% (*n* = 2) affirmed the existence of online information, and no off-floor employee provided directions to this information.

57% (*n* = 4) of security personnel provided general information; 57% affirmed that in-casino material was available, and 57% provided directions to the in-casino materials. Regarding online information, 43% (*n* = 3) affirmed the existence of online information, and 29% (*n* = 2) provided directions to this information.

#### Information Received: Observer

The observer recordings indicated that 67% (*n* = 4) of on-floor employees provided general information about RG. 83% (*n* = 5) reported that RG materials were present in the casino, but only 50% (*n* = 6) provided directions to find the materials. 67% (*n* = 4) affirmed the existence of online information, but only 17% (*n* = 1) correctly provided directions for this information.

71% (*n* = 5) of off-floor employees provided general information. Although 86% (*n* = 6) could provide directions to in-casino materials, none could provide directions to online information. Every off-casino floor employee affirmed that in-casino materials were available, but only 43% (*n* = 3) could affirm that information was available online. Observer reports of the interactions with security personnel were identical to those reported by the actor.

#### Customer Service Experience

Employee attentiveness was, on average, neutral (*M* = 4, *SD* = 1.2). 43% (*n* = 6) of customers rated the staff as somewhat attentive, and 21% (*n* = 3) rated staff as somewhat dismissive.

Regarding employee friendliness, customer ratings were positive (*M* = 5.5, *SD* = 0.7), with 64% rating the employees as at least friendly. In Casino #6, customers reported that the security employee was “very nice.” Customers also noted that the on-floor employee encouraged them to seek help if they needed it and even offered to call their supervisor.

Regarding employee judgment or acceptance, ratings were, on average, slightly positive (*M* = 4.5, *SD* = 1.2). 29% of customers rated the employees as accepting, and 21% (*n* = 3) rated them as somewhat judgmental. In Casino #7, a customer noted that the security employee told them that if they do not spend all their money gambling, they should give the rest of their money to the employee. The actor rated this experience as accepting and somewhat attentive, and the observer rated their experience as somewhat dismissive and judgmental. In Casino #6, both customers reported that staff were somewhat accepting and somewhat knowledgeable, noting that the on-floor employees seemed to genuinely care about their wellbeing.

Responses regarding employee knowledge about RG were typically neutral (*M* = 4.07, *SD* = 1.6). 29% (*n* = 4) of customers rated the employees as somewhat knowledgeable, and 14% (*n* = 2) of customers rated the employees as somewhat ill-informed. In Casino #5, a customer rated an off-floor employee as ill-informed, noting that the employee told them they would be “put on a list” if they “gambled too much.”

## Discussion

The two present studies documented customer experiences when requesting RG information from all casinos within a southeastern, moderately sized metropolitan area market. Casinos were hypothesized to successfully execute their mandated responsibility to provide the requested information with differences in responses possibly being related to employee role at the property. Of the completed calls to the casinos’ customer service phone numbers, the accessibility of RG information was unreliable, and the experience suggested non-compliance with jurisdictional requirements. As for interactions with employees at the casinos, the employee role appeared to influence the information provided, and the low likelihood of a customer’s questions about RG information being answered did not reliably fulfill the regulatory requirements or the spirit of CSR. There were some positives in these experiences. It was likely that an on-floor employee would provide adequate explanations of RG. Off-floor employees were prepared to provide customers with information on the location of in-casino RG materials while demonstrating less knowledge of safe gambling practices. Regarding security personnel, required commitments to RG fueled an expectation that greater numbers would have competently delivered the requested information. Despite the presence of RG information on each property website, requests for such information through both telephone calls and casino visits were disappointingly uninformative.

Calling the casino’s customer service phone number was inconsistently successful and frequently unhelpful. When information was provided, it was often in a dismissive manner. Over the telephone, customers were often transferred between customer service representatives without gaining information and, on average, placed on hold for half of the total call duration. Even after waiting, information was still unobtainable in some instances, and calls were sometimes abruptly disconnected. Previous research suggests that for simple tasks, telephone calls are more effective and task oriented than face-to-face interaction (Kira et al., 2009). Telephone-based RG assistance is an accessible modality for individuals to gather knowledge on RG resources, such as loved ones of those experiencing gambling harm as 97% of Americans own a cellphone (“Mobile Fact Sheet,” 2024). allowing them to contact businesses and facilities at any point in time (Lepp et al., 2013). As such, it is paramount for casinos to provide accurate information and a positive customer experience across such an accessible and task-oriented mode of communication. Unfortunately, the present project found that customers are likely to end a call without desired information and with the impression that the casino operator does not value RG.

Requests for information during casino visits resulted in friendly employee responses; however, the quality of the provided information was inconsistent. Customers noted that employees appeared to care about their well-being and perceived them as attentive during most interactions. However, less than half of the employees in each role affirmed that online resources were available and even fewer provided directions to the information. These findings were surprising, considering every casino provided information on their respective websites and state regulatory agency websites (e.g., https://www.msgamingcommission.com/; https://www.southlandcasino.com/casino/responsible-gaming/).

These present findings raise the question of whether casino employee training regarding RG is inadequate, poorly executed, or not effectively translated to day-to-day operations. It has been shown that employee training increases knowledge of gambling harms and help services (Beckett et al., 2020; Giroux et al., 2008; LaPlante et al., 2012), and employees have reported feeling confident in assisting customers who approach them for assistance (Beckett et al., 2020; Hing & Nuske, 2012). Despite mandated annual training (Mississippi Gaming Control Act, 1990, 2020), very few employees within the current studies had the necessary knowledge of their casino’s online commitment to RG or resources to prevent gambling harm. Previous research has shown that casino employees receive different levels of RG training, with customer-facing employees reporting lower levels of RG training than employees in other roles (e.g., maintenance, security officers; Christensen et al., 2022). Logically, having customer-facing employees familiar with the property’s web presentation of legally mandated information should not be difficult, although current employee training practices may not value it or may provide varying levels of training based on employee role. Considering public CSR commitments that promote RG from each property in this sample and the broad accessibility of this information, it is disappointing that employees were unaware of resources. Reliable CSR efforts have been found to increase employee commitment to business operations (Beckett et al., 2020; Lee et al., 2013) and customer satisfaction in gambling venues (Abarbanel et al., 2018; Beckett et al., 2020); however, the underwhelming provision of online information from these operators suggests a failure to embrace CSR.

The current secret shopper studies had several limitations. First, the small sample size of casinos did not allow for a comparison of results between casinos located in jurisdictions with mandated RG programs and those located in jurisdictions without RG programs. Properties in other locations may have unique CSR commitments or differing regulations within their jurisdictions. Despite the restriction to one market, small markets represent most casino operations in the US (American Gaming Association, 2023). Future studies would benefit from understanding how customer experiences differ based on varying regulations across jurisdictions. Second, a limited number of employees were approached or contacted over the telephone. Staff training may occur annually and not during onboarding, so recently hired employees may have been contacted before they received training. Therefore, the employees contacted might not generalize to the entire staff at each property or from other markets. Third, it is unknown whether the results were due to gaps in RG-specific training or from larger organizational training deficiencies (e.g., general customer service training). Our methods did not isolate these two areas of training, and consequently, RG training and general customer service provision may have been conflated. While it is difficult to conclude whether the lack of RG knowledge may be a product of deficient RG-specific training or general customer service training, these are both barriers to accessing RG information. Fourth, the way that customers approached employees may have caught them by surprise. Although employees have reported feeling confident in assisting customers who approach them for assistance, they also report that these interactions rarely happen (Beckett et al., 2020; Hing & Nuske, 2011b, 2012; Riley et al., 2023). Perhaps the help-seeking customers surprised employees, and employee responses were consequently less articulate.

## Conclusion

In conclusion, our team’s experience was that most casino operators did not adhere to their legal or social responsibility to protect customers from gambling harm. The Reno Model emphasizes that preventing gambling harm and gambling disorder involves interactions between multiple stakeholders, where governments enact legislation requiring operators to implement empirically supported RG programs (Shaffer et al., 2019, 2020). Only after being supported and educated by governments and operators can customers truly develop an informed decision to gamble and access resources if they experience gambling harm (Shaffer et al., 2020). Furthermore, a core component of successful CSR implementation is productive communication between businesses and customers, where information is provided clearly and effectively (Camilleri, 2018; Kocurikova et al., 2024). Customers and employees will only embrace CSR efforts if operators take the initiative to endorse empirically supported RG policies. Until casino operators follow through with their CSR commitments and/or governments hold them accountable for upholding their legal obligations, RG programs will likely remain ineffective in protecting customers from gambling harm.

## Electronic Supplementary Material

Below is the link to the electronic supplementary material.

Supplementary Material 1

## Data Availability

Data will be made available upon reasonable request.
