# Supplementary material for: Accessing Responsible Gambling Information from Casinos: Two Secret Shopper Studies
Source: J Gambl Stud. 2025 May 24;41(4):1597–613. doi: 10.1007/s10899-025-10396-w (PMC12657571; doi:10.1007/s10899-025-10396-w)
Supplement: Supplementary file 1 — Supplementary Material 1 [file 10899_2025_10396_MOESM1_ESM.docx]

**Supplementary Materials for**

**Accessing Responsible Gambling Information from Casinos: Two Secret Shopper Studies**

*Journal of Gambling Studies*

Supplemental Material 1.

*Telephone Call and In-Person Script*

1. “Hi, can you give me general information about responsible gambling?”
2. “Are there any materials in the casino?”
   1. “Like brochures or pamphlets?”

2a. “Where can I find them in the casino?”

1. “Is there any information online?”

3a. “Where can I find it online?”

1. (*For telephone calls*) “Can you mail me any of the materials?”

4a. (*For telephone calls*) If yes, provide an address from the list.

1. “What other information can you suggest for me?”

“Thank you for your time.”

| \| **Supplemental Table 1**  *Call information from the first and second call to each of the six casino properties.* \| \| \| \| \| \| --- \| --- \| --- \| --- \| --- \| \| Casino \| Call Number \| Call Duration \| Time on Hold \| Employee \| \|  \|  \| Min. \| Min. \| *n* \| \| Casino 1 \| 1^st^ \| 3.05 \| 0.53 \| 2 \| \| Casino 1 \| 2^nd^ \| 8.30 \| 4.90 \| 2 \| \| Casino 2 \| 1^st^ \| 2.00 \| 0.62 \| 2 \| \| Casino 2 \| 2^nd^ \| 1.60 \| 0.30 \| 2 \| \| Casino 3 \| 1^st^ \| 9.73 \| 6.03 \| 3 \| \| Casino 3 \| 2^nd^ \| 5.00 \| 2.10 \| 1 \| \| Casino 4 \| 1^st^ \| 3.00 \| 0.85 \| 1 \| \| Casino 4 \| 2^nd^ \| 2.00 \| 0.66 \| 4 \| \| Casino 5 \| 1^st^ \| 10.0 \| 5.61 \| 2 \| \| Casino 5 \| 2^nd^ \| 2.23 \| 0.12 \| 2 \| \| Casino 6 \| 1^st^ \| 5.62 \| 2.76 \| 2 \| \| Casino 6 \| 2^nd^ \| 2.33 \| 0.92 \| 2 \| \|  \|  \| *M* (*SD*) \| *M* (*SD*) \| *M* (*SD*) \| \|  \| Total *n* = 12 \| 4.6 (3.13) \| 2.12 (2.19) \| 2.08 (0.80) \| \| *Note.* Call durations refer to the total duration of the telephone call. Time on hold refers to the total amount of time research assistants were placed on hold throughout the telephone call. The final column displays the number of employees each research assistant interacted with throughout the telephone call. \| \| \| \| \| |
| --- | --- | --- | --- | --- | --- | --- | --- | --- | --- | --- | --- | --- | --- | --- | --- | --- | --- | --- | --- | --- | --- | --- | --- | --- | --- | --- | --- | --- | --- | --- | --- | --- | --- | --- | --- | --- | --- | --- | --- | --- | --- | --- | --- | --- | --- | --- | --- | --- | --- | --- | --- | --- | --- | --- | --- | --- | --- | --- | --- | --- | --- | --- | --- | --- | --- | --- | --- | --- | --- | --- | --- | --- | --- | --- | --- | --- | --- | --- | --- | --- | --- | --- | --- | --- | --- | --- | --- | --- | --- | --- |
